# Impact of Prior Therapeutic Opioid Use by Emergency Department Providers on Opioid Prescribing Decisions

**DOI:** 10.5811/westjem.2016.8.30965

**Published:** 2016-09-29

**Authors:** Adam C. Pomerleau, Jeanmarie Perrone, Jason A. Hoppe, Matthew Salzman, Paul S. Weiss, Lewis S. Nelson

**Affiliations:** * Emory University School of Medicine, Department of Emergency Medicine, Atlanta, Georgia; † University of Pennsylvania, Perelman School of Medicine, Department of Emergency Medicine, Philadelphia, Pennsylvania; ‡ University of Colorado, Department of Emergency Medicine, Aurora, Colorado; § Rocky Mountain Poison and Drug Center, Denver, Colorado; ¶ Rowman University, Cooper Medical School, Department of Emergency Medicine, Camden, New Jersey; || Emory University, Rollins School of Public Health, Department of Biostatistics and Bioinformatics, Atlanta, Georgia; # New York University School of Medicine, Department of Emergency Medicine, New York, New York

## Abstract

**Introduction:**

Our study sought to examine the opioid analgesic (OA) prescribing decisions of emergency department (ED) providers who have themselves used OA therapeutically and those who have not. A second objective was to determine if OA prescribing decisions would differ based on the patient’s relationship to the provider.

**Methods:**

We distributed an electronic survey to a random sample of ED providers at participating centers in a nationwide research consortium. Question topics included provider attitudes about OA prescribing, prior personal therapeutic use of OAs (indications, dosing, and disposal of leftover medication), and hypothetical analgesic-prescribing decisions for their patients, family members, and themselves for different painful conditions.

**Results:**

The total survey population was 957 individuals; 515 responded to the survey, a 54% response rate. Prior personal therapeutic OA use was reported in 63% (95% CI = [58–68]). A majority of these providers (82%; 95% CI = [77–87]) took fewer than half the number of pills prescribed. Regarding provider attitudes towards OA prescribing, 66% (95% CI = [61–71]) agreed that OA could lead to addiction even with short-term use. When providers were asked if they would prescribe OA to a patient with 10/10 pain from an ankle sprain, 21% (95% CI = [17–25]) would for an adult patient, 13% (95% CI = [10–16]) would for an adult family member, and 6% (95% CI = [4–8]) indicated they themselves would take an opioid for the same pain. When the scenario involved an ankle fracture, 86% (95% CI = [83–89]) would prescribe OA for an adult patient, 75% (95% CI = [71–79]) for an adult family member, and 52% (95% CI = [47–57]) would themselves take OA. Providers who have personally used OA to treat their pain were found to make similar prescribing decisions compared to those who had not.

**Conclusion:**

No consistent differences in prescribing decisions were found between ED providers based on their prior therapeutic use of OA. When making OA prescribing decisions, ED providers report that they are less likely to prescribe opioids to their family members, or themselves, than to an ED patient with the same painful condition.

## INTRODUCTION

According to the 2011 National Hospital Ambulatory Medical Care Survey (NHAMCS), pain-related complaints accounted for five of the top 10 principal reasons patients sought care in the emergency department (ED).^1^ Consequently, ED providers are high-volume prescribers of nonprescription analgesics, such as acetaminophen, non-steroidal anti-inflammatory drugs, and of opioid analgesics (OA). Examination of NHAMCS data between 2001–2010 showed an increase from 20.8% to 31% in OA prescribing during ED visits, while the rate of prescriptions for non-opioid analgesics over the same period was unchanged.^2^ Prescribing rates for OA by different specialties between 2007–2012 noted a downward trend by emergency medicine prescribers.^3^ Despite this trend, the absolute number of patients receiving an OA prescription remains high, with emergency physicians providing 12.5 million OA prescriptions in 2012.^3^ However, the number of pills per prescription is low and the opioid formulation chosen is almost exclusively short acting.^4^

Over the past decade, there has been widespread recognition of the adverse effects and risks associated with OA. Even when prescribed for their intended therapeutic benefit, a concerning percentage of patients will develop an opioid use disorder and others will overdose and suffer from consequential respiratory depression. Although ED providers are not primarily responsible for the current epidemic of opioid-related addiction and overdose, all prescribers have been encouraged to examine and rationalize their prescribing decisions.^5^

Prior research shows that decisions to prescribe opioid medications are highly individualized; different providers will make different decisions based on the same information.^6^ Hence, a better understanding of factors underlying prescriber variability may help identify strategies that promote meaningful modification of their prescribing practices. One factor that could affect prescribing decisions, and that has not yet been examined in the literature, is a provider’s personal history of taking OA therapeutically to treat his or her own pain.

Our study sought to examine the OA prescribing decisions of ED providers who have used OA therapeutically compared to those who have not. A second objective was to study the reported prescribing decisions to patients compared to family members with the same painful conditions. We hypothesized that providers who themselves used OA would be less likely to prescribe to their patients and that providers would be less likely to prescribe to family members than to patients.

## METHODS

### Study Design and Setting

This was a multi-center, cross-sectional, web-based survey of 957 ED providers at seven participating centers. The study was conducted between August 2014 and October 2014. Eligible providers included attending physicians, emergency medicine resident physicians, and advanced practice providers (nurse practitioner or physician assistant) who work in the ED. There were no exclusion criteria. Potential respondents were invited to complete a web-based questionnaire via email.

### Selection of Participants

We used the Prescribing Opioids Safely in the Emergency Department (POSED) Research Consortium to conduct the study.^4^ The consortium is comprised of 30 primarily academic medical centers located in 20 states, spanning all four regions of the country, with over two million annual ED visits. A random cluster sample of seven centers was selected from among the 30 total POSED centers. All of the selected centers in the sample are affiliated with an emergency medicine residency program.

All subjects who participated in the study provided informed consent. Respondents completed the survey anonymously. The study protocol was reviewed and approved by the coordinating center’s institutional review board.

### Survey Content and Administration

We developed the study questionnaire in accordance with methods outlined by Burns et al.^7^ The initial questionnaire was written by the investigators and then iteratively developed through feedback solicited from expert colleagues as well as a biostatistician for purposes of item generation and improving structure. Question topics included prior personal therapeutic use of OAs (indications, dosing, and disposal methods of leftover medication), and the type of pain medication providers would prescribe or recommend to their patients, friends, family members, and themselves for two common, painful conditions (ankle sprain and ankle fracture). Additional questions addressed attitudes towards OA prescribing and demographic information. The survey instrument was pilot tested using emergency providers with a similar demographic to the potential respondents to improve question clarity and assist with item reduction. Formal psychometric testing of the questionnaire was not performed.

The survey questionnaire was hosted online and administered using FluidSurveys (http://www.fluidsurveys.com). We identified points of contact (POCs) for each participating center to coordinate distribution of email announcements and to determine the total number of eligible providers at each center. An email announcing the study was sent to potential respondents at each center by the POC, followed several days later by a second email containing a link to the questionnaire. Three reminder emails were sent over the subsequent month to encourage participation. In addition, we offered a nominal incentive to survey respondents in the form of a raffle sweepstakes for a gift card. The raffle database was independently administered from the main study database with no link between the two.

### Data Analysis

The survey collected information on respondents’ attitudes and hypothetical prescribing decisions using a five-point Likert scale. To facilitate analysis in the presence of sparse tables and zero-cells, we collapsed the Likert scale responses into two likelihood groups: Likely (respondents who indicated they were likely or very likely to prescribe) and Unlikely/Neutral (respondents who indicated that they were neutral, unlikely or very unlikely to prescribe). Respondents were also classified into groups who would prescribe an opioid (alone or in combination with other pain medications) and who would not prescribe opioids at all. Data regarding prescribing decisions for different types of patients were stratified using a question about whether respondents would rather over-prescribe and risk misuse of OA or rather underprescribe and risk under-treating pain. Although the data were not complete for every respondent, we used all available data for each analysis. Any missing data were handled using pairwise elimination. We used a simple adjustment to the sampling weights to account for overall survey non-response. All analyses were completed in SAS 9.3 (Cary, NC) using the specialized survey procedures to account for the cluster sampling design. These procedures account for the clustering within the centers by adjusting the chi-square statistic to properly reflect the loss in precision that comes from the increased homogeneity in the observations within a center.

## RESULTS

### Characteristics of Study Subjects

There were 957 eligible ED providers total from the seven selected centers; 515 responded to the survey invitation for an overall response rate of 54%. Twenty-four respondents did not consent to participate in the study, and 48 responses were excluded for insufficient data, leaving 443 responses included for analysis. Demographic data are presented in Table 1. Data were not available regarding the demographics of non-respondents. There was no indication of bias due to variations in response patterns between respondents.

### Personal Therapeutic Use of OA

Sixty-three percent (95% CI = [58–68]) of respondents reported prior personal therapeutic OA use. A majority of these providers (82%; 95% CI = [77–87]) took fewer than half the number of tablets prescribed.

### OA Prescribing Decisions by Patient Type

The figure summarizes the frequencies respondents would prescribe or recommend an OA for different types of patients with an ankle sprain or ankle fracture. Among all respondents, 21% (95% CI = [17–25]) indicated they would prescribe an opioid to an adult patient with an ankle sprain reporting 10/10 pain, while 6% (95% CI = [4–8]) indicated they themselves would take an opioid for the same pain. Similarly, 86% (95% CI = [83–89]) indicated they would prescribe an opioid to an adult patient with an ankle fracture reporting 10/10 pain, while 52% (95% CI = [47–57]) indicated they themselves would take an opioid for the same pain. Thirteen percent (95% CI = [10–16]) would prescribe an OA to an adult family member with an ankle sprain and 75% (95% CI = [71–79]) would do the same for an adult family member with an ankle fracture. Rates were lower for a teenage family member, with 4% (95% CI = [2–6]) prescribing OA for an ankle sprain and 42% (95% CI = [37–47]) for an ankle fracture.

### Attitudes Towards OA Prescribing

When asked about their attitudes towards OA prescribing, 92% (95% CI = [89–95]) of respondents agreed it is important for ED providers to consider the public health effects of prescribing OA. In addition, 76% (95% CI = [72–80]) agreed that ED prescriptions are a source of OA that are used non-medically or diverted. Sixty-six percent (95% CI = [61–71]) agreed that OA could lead to addiction even with short-term use. Finally, 43% (95% CI = [38–48]) indicated they would rather over-treat patients in pain with OA to avoid under-treating a single patient with significant pain, while 57% (95% CI = [52–62]) indicated they would rather under-treat patients in pain with limited or no OA to avoid causing addiction, dependence, or overdose in a single patient.

### Comparison of OA Prescribing Decisions Among Providers

Table 2 summarizes the number of respondents who would prescribe OA to a patient complaining of 10/10 pain from an ankle sprain comparing those respondents who have a history of personal therapeutic OA use and those who do not. Table 3 is a similar comparison of respondents except the patient has 10/10 pain from an ankle fracture instead of an ankle sprain.

Table 4 summarizes the number of respondents who would prescribe OA to a patient complaining of 10/10 pain from an ankle sprain comparing providers who indicated they would rather over-treat with OA to avoid under-treating a patient in pain to providers who would rather under-treat with OA to avoid opioid misuse. Table 5 is a similar comparison of respondents except the patient is complaining of 10/10 pain from an ankle fracture.

## DISCUSSION

One objective of this study was to assess whether prior therapeutic OA use was associated with a clinician’s current OA prescribing decisions. The majority of ED providers in this study reported prior personal therapeutic OA use and the vast majority of these providers took fewer than half the number of tablets prescribed. Tables 2 and 3 show that more respondents who had personally taken opioids indicated they would prescribe OA compared to those who had not. However, it is important to note that the confidence intervals overlap for eight of the 10 comparisons, which prohibits drawing a general conclusion of a difference based on prior OA use. The absence of an observed difference could mean that providers are truly not influenced by their own experience using OA therapeutically when making prescribing decisions. It is also possible the influence of prior OA use on prescribing decisions is more nuanced than could be detected based on our questionnaire. We did not ask providers whether they had a favorable or negative experience with the OA they had used; providers with favorable experiences may be more likely to prescribe OA while those with negative experiences may be less likely to prescribe. The personal experiences using OA therapeutically may influence provider beliefs about the safety, efficacy, and risk-versus-benefit relationship of these drugs. Lastly, it is also possible that the sample size was simply not large enough to show a true difference.

A second objective of this survey was to determine if OA prescribing would differ based on the patient’s relationship to the prescriber. The figure illustrates a consistent trend found among respondents; when treating the same pain, more ED providers indicated they would prescribe an OA to one of their patients more frequently than to a family member (adult or teenage) or to themselves. This is demonstrated by a three-fold relative difference in prescribing an OA for a patient with an ankle sprain compared to self-use for the same indication (21%; 95% CI = [17–25] vs. 6%; 95% CI = [4–8]). These data suggest that ED providers may treat themselves and their family members differently than how they treat their patients.

Reasons for this observed difference in practice can only be speculated. Presumably there is no intrinsic difference in the risk-versus-benefit profile for OA use in a given individual based solely on whether that person is related to a provider. Inferred from our data is that ED providers might be more cautious prescribing OA to family members or themselves out of concerns about harms or lack of benefit over other types of pain medications. Our finding that the majority of respondents were concerned that even short-term use of opioids can trigger addiction supports this inference.

Other reasons for differential prescribing by population might include the pressure to meet patient expectations or maintain high patient-satisfaction scores. It has been suggested that the priority placed on achieving high patient satisfaction scores could carry unintended consequences of driving inappropriate OA prescribing.^8^ Evidence to address this issue thus far is mixed. One study of patients with painful conditions at a single ED found a significant association between patient satisfaction and reduction in their level of pain.^9^ Another retrospective study found no association between Press Ganey satisfaction scores and receipt of OA while in the ED.^10^ There are no conclusive data yet to define whether provider satisfaction scores drive OA prescribing decisions. In our experience, certain clinicians likely prescribe more OA than is typically required in order to avoid suboptimal analgesia in a few or to avoid the need for unscheduled follow up for pain.

It is notable that OA were consistently more frequently prescribed by providers when treating ankle fracture compared to ankle sprain (Figure). The clinical scenarios presented in the survey questionnaire described a patient with 10/10 pain with either an ankle fracture or ankle sprain. Despite the similar pain score, there were large differences in prescribing decisions between fracture and sprain, when in reality the pain experienced by patients with these diagnoses may be very similar. This difference may reflect providers’ bias towards fracture being considered an intrinsically more painful condition and a prioritization of this assessment over the reported pain scores.

Tables 2–5 reveal that there were larger differences in the frequencies of OA prescribing for patients with an ankle sprain than with an ankle fracture. Table 2 shows that 21% of respondents would prescribe an opioid to an adult patient with an ankle sprain, while Table 3 shows 86% of respondents would prescribe an opioid to an adult patient with an ankle fracture. This result suggests that most ED providers agree ankle fracture is an appropriate indication to treat using OA. Table 4 offers evidence of increased variability of OA use between ED providers for the “softer” indication of ankle sprain showing a clear pattern of providers who would rather over-treat pain prescribing OA more often than those who would rather under-treat pain. Table 5 shows less variability of OA prescribing for ankle fracture based on the difference in attitude towards prescribing. If this attitudinal disparity towards prescribing OA can produce such a difference between providers, it begs the larger question about deciding what “appropriate prescribing” of OA truly means.

The majority of ED providers surveyed believe they are important sources of opioids used non-medically, that even short-term OA use risks addiction, and that ED prescribing decisions should be made with consideration of public health implications. One possible explanation for these views may have to do with the types of patients and situations commonly encountered in the ED. ED providers prescribe analgesics for large numbers of patients with painful conditions, the majority of whom have acute, generally self-limited pain. In addition, a substantial number of patients with intermittent and chronic pain syndromes seek care in the ED.^11^ ED providers must be facile with properly targeting the use of safe and effective analgesics for outpatient use following discharge from the ED. This task is complicated by the lack of an ongoing provider-patient relationship with most patients. Furthermore, this lack of an ongoing relationship, and the ED’s “open door” nature, make the ED vulnerable to individuals intent on obtaining opioids for aberrant uses. Prior literature confirms EDs as locations targeted by these individuals. One statewide study showed that 88% of ED providers reported seeing at least one provider-shopping patient per week.^12^–^15^ It seems reasonable to think ED providers who are concerned about public health and the contribution of their prescribing to the opioid epidemic prescribe less in the same situations than those without such concerns. While our data could not be used to directly assess any such associations, future investigations designed to test this possibility would likely provide valuable information.

Finally, it is widely postulated that teenagers are at a higher risk for developing opioid use disorder than adults; recent evidence suggests that use of OA before 12^th^ grade is associated with future opioid use disorder.^16^ It is therefore interesting that ED providers in our study would prescribe opioids less frequently to teenage patients compared to adult patients, and even more judiciously to teenage family members compared to adult family members. This may reflect a different risk-benefit profile for adult and teenage populations. Of note is that respondents more frequently opted not to prescribe opioids for teenage family members compared to their teenage patients for ankle sprain. This likely again reflects a differential risk-benefit analysis in family versus patients.

## LIMITATIONS

We did not collect direct measurements of behaviors, so we do not know how the study population would actually perform in real-world situations. Formal psychometric analysis of the questionnaire was not performed and respondents may interpret some questions differently due to potentially unclear or misunderstood language. This survey may be confounded by social desirability bias; respondents may have unknowingly over-reported what they consider more socially desirable behaviors and under-reported what they interpreted as less desirable behaviors. In addition, the results pertaining to personal therapeutic use of OA may also be affected by recall bias on the part of respondents. The generalizability of the results may be affected by the overall response rate and the preponderance of academic medical centers. There is also potential for selection bias insofar as only providers from centers participating in the POSED consortium comprised the study population. However, we took several steps in our analysis to mitigate what might be considered a low response rate in order to maximize the representativeness of our results. Our analyses properly adjusted for cluster. Standard analytical methods require an underlying simple random sample design; our complex design was chosen based on ease of implementation and reduction in cost. A simple random sample would have required a list of participating physicians and would have been much more difficult to implement in real life. A less rigorous approach (e.g., sending a survey to every provider in the network) would have provided a more biased estimate and the representativeness of the convenience sample would have been impossible to determine. Detailed demographic information regarding the entire population across all the centers is unknown; non-response bias could also confound the results. Lastly, while the results do not show statistical differences in prescribing decisions, we do think the results are of clinical importance. The results suggest providers’ personal experiences with OA can influence prescribing decisions. Moreover, the differences in prescribing decisions for patients compared to family members suggests that providers are prescribing OA to patients despite having concerns about drug-related risks to which they would not want to expose their family members. We find this telling of strong factors favoring OA prescribing among ED providers that have yet to be fully elucidated.

## CONCLUSION

No consistent differences in prescribing decisions were found between ED providers based on their prior therapeutic use of OA**.** Providers were more likely to prescribe OA for severe pain due to ankle fracture compared to ankle sprain. When making OA prescribing decisions, ED providers report that they are less likely to prescribe opioids to their family members, or themselves, than to an ED patient with the same painful condition.

## Figures and Tables

**Figure:**
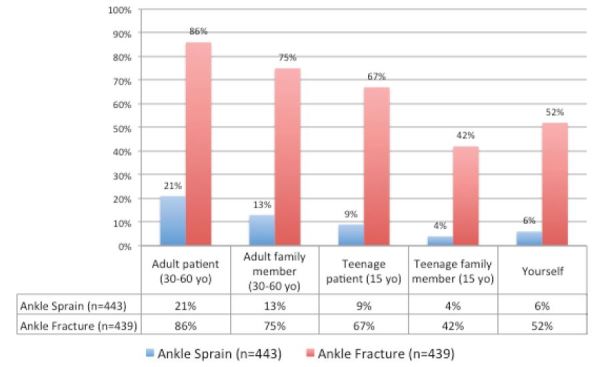
Frequency of opioid analgesic prescription by patient type for ankle sprain and fracture.

**Table 1:** Number and percentage of respondents with different demographic characteristics and prior therapeutic opioid analgesic use.

Characteristic	Number (%)
Male (n = 412)	220 (53)
Role in ED (n = 419)	
Attending	207 (49)
Resident	170 (41)
Advanced practice provider	42 (10)
Years working in ED (n = 417)	
< 1 year	52 (13)
> 1 – < 5 years	164 (39)
> 5 – < 10 years	53 (13)
> 10 – < 20 years	85 (20)
> 20 years	63 (15)
Location of ED* (n = 420)	
Rural	8 (2)
Suburban	24 (6)
Urban	410 (98)
ED practice setting* (n = 420)	
Academic	409 (97)
Community	71 (17)
VA/military	16 (4)
Prior therapeutic OA use (n=420)	265 (63)

*ED,* emergency department; *OA,* opioid analgesic

* Cumulative responses exceed 100%; respondents were asked to identify all types of ED practice settings in which they worked.

**Table 2:** Percentage of respondents who would prescribe an opioid analgesic (OA) to a patient complaining of 10/10 pain from an ankle sprain by history of personal therapeutic OA use.

Ankle sprain: patient type	Overall % [95% CI] n=443	Personal OA yes % [95% CI] n=265	Personal OA no % [95% CI] n=157
Adult patient (30–60 years)	21 [17–25]	24 [19–29]	16 [10–22]
Adult family member	13 [10–16]	15 [11–19]	11 [6–16]
Teenage patient (15 years)	9 [6–12]	10 [6–14]	5 [2–8]
Teenage family member	4 [2–6]	5 [2–8]	2 [0–4]
Yourself	6 [4–8]	7 [4–10]	4 [1–7]

*CI,* confidence interval

**Table 3:** Percentage of respondents who would prescribe an opioid analgesic (OA) to a patient complaining of 10/10 pain from an ankle fracture, by history of personal therapeutic OA use.

Ankle fracture: patient type	Overall % [95% CI] n=439	Personal OA use % [95% CI] n=265	No personal OA use % [95% CI] n=157
Adult patient (30–60 years)	86 [83–89]	89 [85–93]	85 [79–91]
Adult family member	75 [71–79]	78 [73–83]	72 [65–79]
Teenage patient (15 years)	67 [63–71]	73 [68–78]	59 [51–67]
Teenage family member	42 [37–47]	48 [42–54]	35 [28–42]
Yourself	52 [47–57]	60 [54–66]	41 [33–49]

*CI,* confidence interval

**Table 4:** Percentage of respondents who would prescribe an opioid analgesic to a patient complaining of 10/10 pain from an ankle sprain, by whether they would rather over-treat or under-treat pain using opioids.

Ankle sprain: patient type	Overall % [95% CI] n=443	Over-treat pain % [95% CI] n=178	Under-treat pain % [95% CI] n=233
Adult patient (30–60 years)	21 [17–25]	32 [25–39]	12 [8–16]
Adult family member	13 [10–16]	23 [17–29]	8 [5–11]
Teenage patient (15 years)	9 [6–12]	13 [8–18]	4 [1–7]
Teenage family member	4 [2–6]	7 [3–11]	2 [0–4]
Yourself	6 [4–8]	12 [7–17]	2 [0–4]

*CI,* confidence interval

**Table 5:** Percentage of respondents who would prescribe an opioid to a patient complaining of 10/10 pain from an ankle fracture by, whether they would rather over-treat or under-treat pain using opioids.

Ankle fracture: patient type	Overall % [95% CI] n=439	Over-treat pain % [95% CI] n=178	Under-treat pain % [95% CI] n=233
Adult patient (30–60 years)	86 [83–89]	88 [83–93]	88 [84–92]
Adult family member	75 [71–79]	80 [74–86]	73 [67–79]
Teenage patient (15 years)	67 [63–71]	74 [68–80]	64 [58–70]
Teenage family member	42 [37–47]	53 [46–60]	35 [29–41]
Yourself	52 [47–57]	61 [54–68]	45 [39–51]

*CI,* confidence interval
